# An Advanced View on Baculovirus *per Os* Infectivity Factors

**DOI:** 10.3390/insects9030084

**Published:** 2018-07-17

**Authors:** Bob Boogaard, Monique M. van Oers, Jan W. M. van Lent

**Affiliations:** Laboratory of Virology, Wageningen University and Research, Droevendaalsesteeg 1, 6708 PB Wageningen, The Netherlands; monique.vanoers@wur.nl (M.M.v.O.); jan.vanlent@wur.nl (J.W.M.v.L.)

**Keywords:** baculovirus, membrane fusion, *per os* infectivity factors, PIF, ODV-E56, ODV-E66, ODV entry complex, protein trafficking, R18 de-quenching assay

## Abstract

Baculoviruses are arthropod-specific large DNA viruses that orally infect the larvae of lepidopteran, hymenopteran and dipteran insect species. These larvae become infected when they eat a food source that is contaminated with viral occlusion bodies (OBs). These OBs contain occlusion-derived viruses (ODVs), which are released upon ingestion of the OBs and infect the endothelial midgut cells. At least nine different ODV envelope proteins are essential for this oral infectivity and these are denoted *per os* infectivity factors (PIFs). Seven of these PIFs form a complex, consisting of PIF1, 2, 3 and 4 that form a stable core complex and PIF0 (P74), PIF6 and PIF8 (P95) that associate with this complex with lower affinity than the core components. The existence of a PIF complex and the fact that the *pif* genes are conserved in baculovirus genomes suggests that PIF-proteins cooperatively mediate oral infectivity rather than as individual functional entities. This review therefore discusses the knowledge obtained for individual PIFs in light of their relationship with other members of the PIF complex.

## 1. Introduction

The *Baculoviridae* form a family of arthropod-specific large double stranded DNA viruses that infect insect larvae. The family is divided into four genera, which reflects the co-evolution of these viruses and the insects they infect. Viruses classified in the genera *Alphabaculovirus* and *Betabaculoviruses* infect the larvae of lepidopteran insect species. *Gammabaculoviruses* infect larvae of hymenopteran insect species and the single known virus classified as *Deltabaculovirus* infects *Culex* mosquito larvae (Reviewed by Williams et al. [1]). The majority of baculoviruses that infect lepidopteran larvae have narrow host ranges, that is, a specific baculovirus infects the larval stage of only one or a few host species. Thus, several baculoviruses have been successfully used as biological control agents for particular species of Lepidoptera that are economic pests in agriculture or forestry. (Reviewed by Szewczyk et al. [2]). For example, Anticarsia gemmatalis multiple nucleopolyhedrovirus (AgMNPV) is used to protect soybean plants against the velvet bean (*Anticarsia gemmatalis*) caterpillar in Brazil [3].

The name baculovirus derives from the Latin word baculum, which refers to the rod-shaped morphology of their nucleocapsids. All baculoviruses have large, circular double stranded DNA genomes that range from 80–180 kbp and encode 90–180 genes [1]. Baculoviruses form two different virion phenotypes: budded viruses (BVs) and occlusion-derived viruses (ODVs). The BV virion type is responsible for systemic spread of the infection within the larvae. Each BV consists of a single nucleocapsid surrounded by an envelope derived from the plasma membrane of a host cell. These BVs display a fusion protein to allow cell entry (either GP64 or F protein). ODVs in contrast are formed in the nucleus of infected cells and consist either of a single (SNPV) or multiple (MNPV) nucleocapsids. ODVs are surrounded by an envelope that is derived from the inner nuclear membrane and contains a number of proteins encoded by baculovirus genes (see below). ODVs are also embedded in a crystalline matrix, forming occlusion bodies (OBs). The OBs produced by Alpha-, Gamma- and Deltabaculoviruses are comprised of a viral protein named polyhedrin, while the OBs produced by Betabaculoviruses are comprised of a different viral protein, named granulin. The structure of the OBs protects the ODVs against detrimental influences from the environment. ODVs are responsible for the horizontal transmission of the virus. When insect larvae eat OB-contaminated food, the OBs disintegrate in the highly alkaline milieu of the larval midgut, which releases ODVs into the gut lumen. ODVs then bind to the microvilli of midgut columnar cells and the viral envelope fuses with the cell membrane, releasing nucleocapsids into the cell. This process of virus entry is mediated by a specific set of proteins in the ODV-envelope, the so-called *per os* infectivity factors (PIFs). For Autographa californica multiple nucleopolyhedrovirus (AcMNPV), Peng and co-workers showed that PIFs form a large protein complex, suggesting that these proteins mediate midgut entry of baculovirus ODVs in a cooperative manner [4,5]. In this review, we summarize current knowledge of PIF-protein structure and function.

## 2. Definition and General Features of PIFs

*Per os* infectivity factors are proteins that are essential for primary infection of the midgut columnar cells as deletions of *pif*-genes inhibit oral infectivity of ODVs. Using AcMNPV, nine ODV-envelope proteins have been identified as PIF (Table 1). Since PIFs are not needed for cell to cell spread of the virus within the insect, the infectivity of BVs is not affected after deletion of a *pif*-gene. As a consequence, *pif* deletion mutants show wildtype characteristics in cultured insect cells. Larvae, however, can only be infected with such mutants when the midgut is bypassed by injecting BVs into the hemocoel [6,7,8]. All known PIF proteins identified to date are encoded by baculovirus core genes, that is, they are present and highly conserved in all sequenced baculovirus genomes across the four genera in the family *Baculoviridae* [9,10] Some PIFs are also found in other invertebrate DNA viruses like nudiviruses, bracoviruses, hytrosaviruses and nimaviruses (Reviewed by Wang et al. [10]). This high level of conservation suggests that PIFs mediate an ancient virus entry pathway.

**Figure 1:**
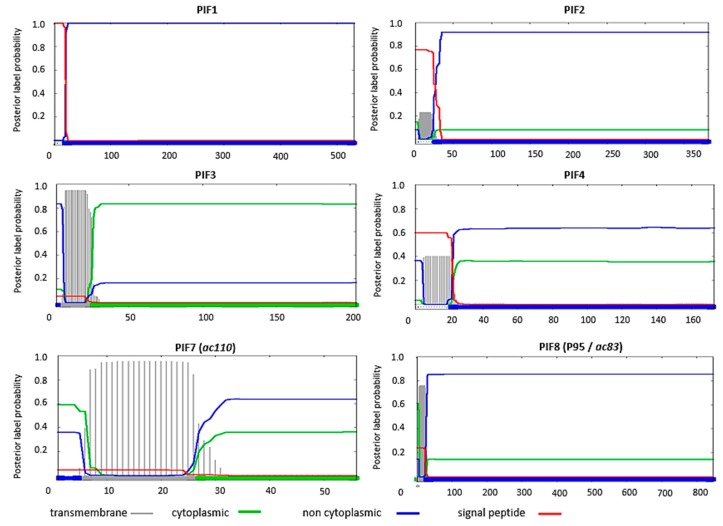
Prediction of the presence of transmembrane domains and signal peptides in *per os* infectivity factors (PIFs) and orientation of these proteins in in an infected host-cell with the Phobius algorithm [17]. On the *y*-axis: the probability for the presence of transmembrane domains (grey), cytoplasmic (green) or non-cytoplasmic (blue) orientation of the amino acid sequence and presence of a signal peptide (red). The final result of the prediction is summarized by the bar at the bottom with the indicated colours. The *x*-axis shows the number of the amino acids of that specific sequence. PIF1, PIF2, PIF3, PIF4, PIF7 (*ac110*) and PIF8 (P95/*ac83*) are predicted to have an N-terminal signal peptide or transmembrane domain.

**Figure 2:**
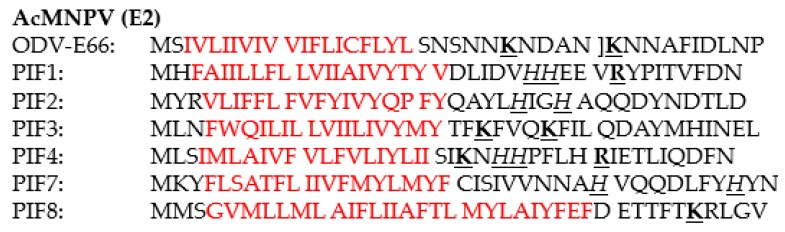
Comparison of the N-terminal transmembrane domains of PIF1, PIF2, PIF3, PIF4, PIF7 and PIF8 with the inner-nuclear membrane sorting motif of ODV-E66. In red: the hydrophobic region; in bold and underlined: the positively charged amino acids lysine (**K**) and arginine (**R**); in italic and underlined the amino acid histidine (**H**) which is only positively charged when protonated.

**Figure 3:**
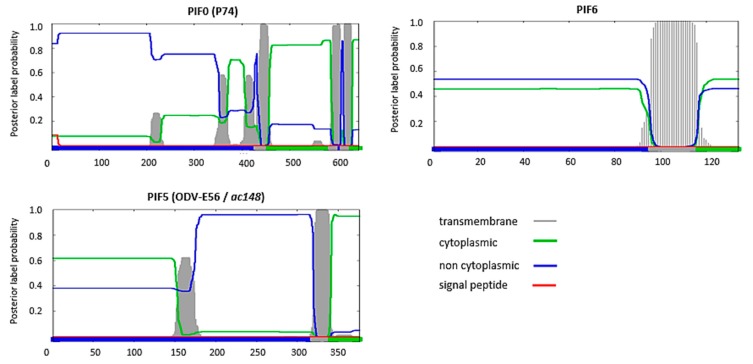
Prediction of the presence of transmembrane domains and signal peptides in PIF0, PIF5 and PIF6 and orientation of these proteins in an infected host-cell with the Phobius algorithm [17]. On the *y*-axis: the probability for the presence of transmembrane domains (grey), cytoplasmic (green) or non-cytoplasmic (blue) orientation of the amino acid sequence and presence of a signal peptide (red). The final result of the prediction is summarized by the bar at the bottom with the indicated colours. The *x*-axis shows the number of the amino acids of that specific sequence.

A feature shared by all PIFs is the presence of highly conserved cysteine residues (Table 1). This high-level of cysteine conservation indicates that sulphate bridges are important for the structural and functional properties of PIFs. *In silico* analysis, using the Phobuis algorithm to find transmembrane domains and signal peptides, revealed N-terminal signal peptides and/or transmembrane helices in six out of the nine PIFs (PIF1 to 4, PIF7 and 8) (Figure 1) [17]. For ODV-E66, another ODV-envelope protein, such an N-terminal domain was shown to be important for its translocation to the inner nuclear membrane [18]. This domain was therefore called the inner nuclear membrane sorting motif (INM-SM) and consists of a hydrophobic domain of about 18 amino acids followed by at least one positively charged amino acid within 4–8 amino acids from the C-terminal end of the hydrophobic sequence. The predicted N-terminal transmembrane domain of the abovementioned PIFs also meets these criteria and the importance of this domain for routing to the inner nuclear membrane has been confirmed for PIF3 and PIF8 (Figure 2) [16,19]. Similar N-terminal regions were not predicted for PIF0, PIF5 and PIF6 (Figure 3). These proteins rather have hydrophobic domains at their C-termini, suggesting an alternative way for routing to the inner nuclear membrane and the ODV-envelope.

## 3. The AcMNPV PIF-Complex

**Figure 4:**
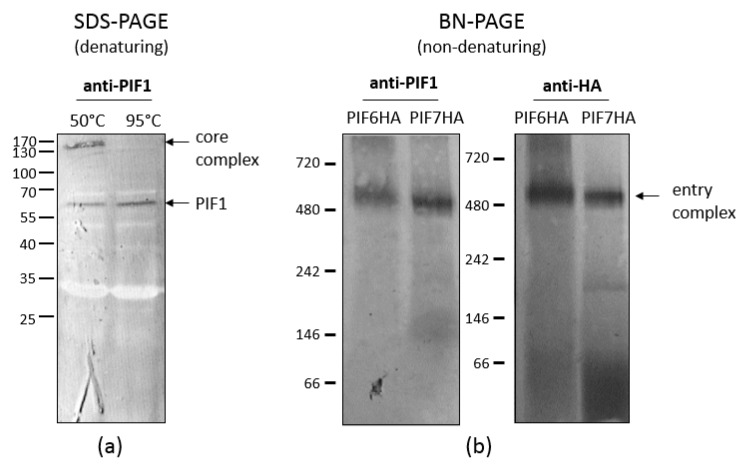
Detection of the stable core complex and the entry complex. (**a**) When isolated occlusion-derived viruses (ODVs) were analysed under denaturing conditions by incubation in Laemmli buffer at 50 °C, the core complex was found as a 170 kDa band by western blot with PIF1 antiserum. Under these conditions, also PIF1 monomers were detected at 60 kDa. When the ODVs were incubated at 95 °C, the core complex dissociated and only PIF1 monomers were found. (**b**) When isolated ODVs were analysed under non-denaturing conditions, the entry complex was detected as a band of approximately 480 kDa with antiserum against PIF1 (left panel) and with anti-HA antibodies against HA-tagged PIF6 (right panel). When analysing ODVs that contain HA-tagged PIF7 with anti-HA antibodies, the entry complex was also found, indicating that PIF7 is part of this complex (right panel).

As shown by Peng et al. [4,5], AcMNPV forms a macromolecular complex in the ODV envelope. So far, seven out of nine PIF proteins have been identified as components of this complex. This so-called ODV entry complex consists of a stable core, formed by PIF1, 2, 3 and 4, to which PIF0, 6 and 8 are more loosely associated. The core of the complex is characterised as stable, since it appears as a 170 kDa band in western blot analysis when ODVs were partially denatured by SDS and reducing agents and incubation at 50 °C (Figure 4a).

When under these conditions the temperature was increased to 95 °C, the core complex completely dissociated into PIF monomers [5]. PIF1, 2 and 3 are essential for formation of the core complex, since the complex was not detected when any of the corresponding genes was deleted [5]. After deletion of *pif4*, however, a smaller complex consisting of PIF1, 2 and 3 was found by western blot analysis [4].

**Figure 5:**
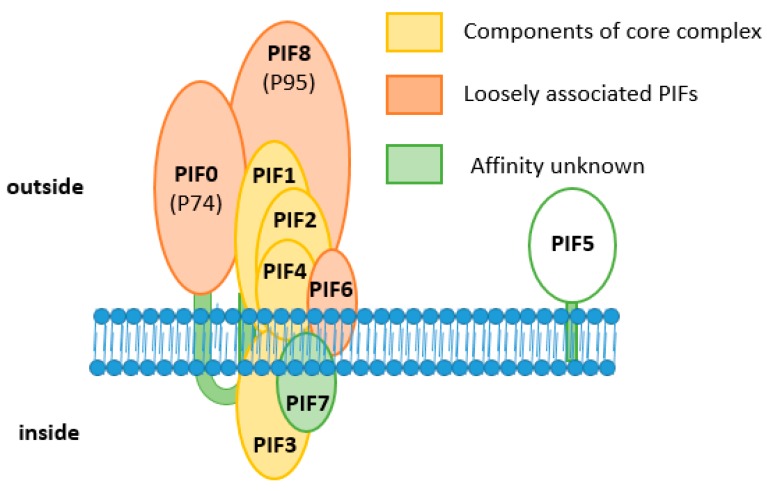
Model of the ODV entry complex. In yellow, the components of the core complex (PIF1, 2, 3 and 4); and in red, the PIFs that associate to the entry complex with lower affinity (PIF0, 6 and 8). PIF7 is depicted in green as this protein was detected in the entry complex under non-denaturing conditions but the affinity of this PIF for the complex is not known. PIF5 does not make part of the entry complex and is depicted in white.

When ODVs were analysed under non-denaturing conditions by blue native-PAGE, the entry complex had an apparent molecular mass of 480 kDa (left panel Figure 4b). This large multi-molecular complex contains, besides the components of the core (PIF1, 2, 3 and 4), also PIF0, 6 and 8 [4,20]. ODV-envelope protein AC110 was recently assigned as PIF7 [15]. To find out whether this PIF is also a component of the large entry complex, we analysed a virus with an HA-tagged PIF7 in the ODV-envelope under non-denaturing conditions. With anti-HA antibodies, we were able to detect the same 480 kDa band as found with PIF1 antiserum (right panel Figure 4b; *unpublished data*). This indicates that PIF7 is a component of the entry complex, which brings us to a total of eight complex-associated PIFs (Figure 5). Apparently, PIF0, 6, 8 (and maybe also PIF7) associated to the core with lower affinity as these PIFs dissociated after (partial) denaturation by treatment with SDS and reducing agents, in contrast to the core components. Only PIF5 appears not to be present in the entry complex and this was confirmed by co-immune precipitations with PIF1 antiserum, in which this PIF was not found as interaction partner of PIF1 [4]. This raises the question whether PIF5 functions independently from the entry complex during primary infection of the midgut. This co-immunoprecipitation study however, resulted in co-precipitation of PIF2, 3, 4, 0, 6 and 8, providing additional evidence for their presence in the entry complex. PIF7 was not found as an interacting partner in this study. AC5 and AC108 also precipitated with PIF1 [4]. AC5 has recently been shown to associate with OBs and it seems not to be involved in oral infectivity or formation of the entry complex [21]. This finding seems not so surprising considering the fact that *ac5* homologs are present only in a subset of the Alphabaculoviruses, in contrast to other *pif* genes. Whether AC108 is involved in either oral infectivity or complex formation still remains to be established.

## 4. PIFs Mediate Viral Entry under Alkaline Conditions in the Midgut

Depending on the insect species, the pH in the larval midgut varies between 10 and 12, which makes it probably the most alkaline environment in nature [22]. Orally ingested OBs dissolve in this alkaline environment, releasing ODVs in the midgut lumen. The ODVs then first encounter the peritrophic matrix. This matrix is composed of chitin, mucopolysaccharides and proteins and mechanically protects the epithelial midgut cells from the food bolus in the gut lumen (reviewed by Hegedus et al. [23]). Some baculovirus species encode metalloproteinases, called enhancins, which degrade the mucin component of the peritrophic matrix [24,25,26]. However, most baculovirus species, including AcMNPV, do not encode such enhancins, while their ODVs still pass the peritrophic matrix. Hence, it is unclear to which extent this matrix is a barrier for ODVs to reach the midgut epithelial cells and whether PIFs are involved in passing this structure as recognized by Rohrmann et al. [27]. PIF8 (P95) is so far, the only PIF for which experimental data support a role in assisting ODVs in passing the peritrophic matrix (see section PIF8 below). 

**Figure 6:**
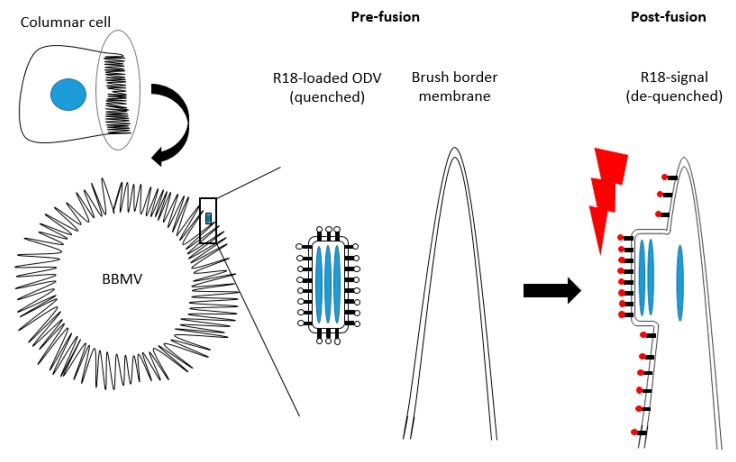
Schematic representation of the R18 de-quenching assay. The fluorescent activity of R18 is quenched initially in the ODV envelope because of the high concentration. Upon membrane fusion, R18 dilutes and gives a fluorescent signal.

Once ODVs have passed the peritrophic matrix, they infect midgut columnar cells via microvilli that form so-called brush borders at the luminal side of these cells. Electron microscopic analysis of the brush border of *Trichuplusia ni* larvae showed numerous nucleocapsids inside microvilli within four hours of inoculation [28,29]. Furthermore, virus-binding assays revealed that ODVs bind to the brush borders in a saturable manner and that binding was significantly reduced upon protease pre-treatment of the brush borders [30]. These findings suggest that the ODVs bind to an as yet unknown proteinaceous receptor on the microvilli. The subsequent fusion between the ODV-envelope and the microvillar cell membrane has been demonstrated by octadecyl rhodamine (R18) de-quenching assays (Figure 6). In this assay, isolated brush border membrane vesicles (BBMV), an in vitro model for the columnar cell’s brush border, were mixed with ODVs that were loaded with high concentrations of the fluorescent dye R18. Due to the high concentration of R18 in the ODV-envelope, the fluorescence of the dye is quenched. However, upon fusion of the envelope with the BBMV, R18 dilutes in the fused membranes which results in de-quenching of the fluorescent signal that then can be recorded [31]. This approach revealed that the fusion between ODVs and BBMV’s occurs at 4 and 27 °C and in a pH-range of 4–11 [30]. Binding and fusion was most efficient at 27 °C under alkaline conditions. Therefore, fusion via the endocytic pathway, as shown for BVs, is less likely as this pathway takes place under acidic conditions [30,32,33].

Microvilli seem to be suitable structures for membrane fusion, as their cylindrical shape and narrow diameter facilitates interactions between membranes. The curvatures create disorder in the membrane, which may facilitate intermixing of (in this case viral and host) membranes during fusion [34]. In addition, the microvilli provide a scaffold for adhesion molecules in which polarized actin filaments create micro-domains that can concentrate adhesion and fusion complexes at the tip of the microvilli [34]. On the side of the virus, PIFs play an essential role in virus entry into the midgut epithelial cells. These proteins are implicated in binding, membrane fusion and further downstream processes like intracellular transport of the nucleocapsids to the nucleus. In the following sections, PIF functions will be discussed. 

## 5. PIFs of the Stable Core of the ODV Entry Complex

### 5.1. PIF1 and 2 Mediate Binding to the Columnar Cell Microvilli

PIF1, 2, 3 and 4 form the stable core in the ODV entry complex. Functionally, PIF1 and PIF2 appear to be involved in binding of ODVs with the microvilli of columnar cells, as has been demonstrated by Ohkawa et al. [11] with R18 de-quenching assays (the technique is explained above). These assays showed that absence of either PIF1 or PIF2 in the ODV envelope resulted in a threefold decrease in ODV binding compared to the wild type. However, once these crippled ODVs had bound, at least half of them was able to fuse with the epithelial membrane, similar as for the wild type virus. This indicates that the absence of either PIF1 or 2 affects ODV binding but not fusion. Live imaging of ODVs with GFP-tagged nucleocapsids also showed that at least a fraction of the ODVs of *pif1*- and *pif2* deletion mutants were still able to bind to the microvilli of freshly isolated epithelial cells [35]. The finding that PIF1 and PIF2 are involved in ODV binding is in accordance with predictions of the Phobius-algorithm, which indicate that the topology of these PIFs is non-cytoplasmic (Figure 1). In this orientation, these PIFs point out from the ODV-envelope after virion envelopment, according to the model of Shi et al. [36], making them available for interaction with host cell receptors [36]. However, as PIF1 and PIF2 are essential for formation of the stable core and therefore the ODV entry complex [5], the observed phenotypes in absence of these PIFs may (at least in part) result from lost interactions with other components of the entry complex. So, whether PIF1 and PIF2 are involved in ODV-binding via direct interactions with host factors or indirect by facilitating formation of the entry complex or may be even both is not clear. 

### 5.2. PIF3 Mediate Oral Infectivity after Binding and Fusion

PIF3, the third component that is essential for formation of the core complex, is not involved in ODV binding and fusion as determined by R18 de-quenching assays [5,11]. It was proposed that PIF3 is involved in oral infectivity in a process downstream from binding and fusion, for example by aiding translocation of the nucleocapsids to the nucleus. This is supported by predictions of the Phobius algorithm, which indicate PIF3 is cytoplasmic oriented in the host cell and thus is probably directed towards the nucleocapsids and would therefore not be available to interact with a host receptor (Figure 1) [36]. Furthermore, Song et al. [37] reported the presence of low levels of PIF3 in the nucleocapsid fraction of HearMNPV ODVs, suggesting an interaction with the nucleocapsid. The finding that *pif3*-deletion does not affect ODV binding and fusion, implies that binding and fusion may also occur in absence of the entry complex as its stable core cannot be formed in absence of PIF3 [5].

Predictions of the Phobius algorithm also indicate that PIF3 has an N-terminal transmembrane domain (Figure 1). Analysis of viruses with PIF3 truncations- and amino acid substitutions revealed that the N-terminal transmembrane domain is crucial for routing to the inner nuclear membrane but that the final incorporation into the ODV-envelope is mediated by other parts of PIF3 [19]. Two PIF3 truncation mutants, in which either twenty or forty amino acids were deleted downstream from the N-terminal transmembrane domain, were routed to the inner nuclear membrane but were not incorporated into the ODV-envelope. The ODVs of these mutant viruses were not orally infectious and is as such phenotypically the same as a *pif3* deletion mutant. Similar results were found when the C-terminal forty amino acids were removed. Two mutants, however, a twenty amino acid C-terminal truncation mutant and a mutant in which the cysteine residue C164 was replaced by a glycine, still showed a low-level of oral infectivity while these mutated forms were also not detected by western blot analysis. Of all mutants, the C162G substitution mutant is particularly interesting as oral infectivity was severely affected, while this form of PIF3 was still incorporated into the ODV-envelope. The C162G mutant is the only PIF3 mutant where loss of oral infectivity can be directly related to the mutation itself and not to side-effects as affected routing. This study demonstrates that it can be difficult to analyse PIF-proteins functions by mutagenesis as this might affect routing to the ODV envelope. 

### 5.3. The Role of PIF1-3 in Host-Range Determination

PIFs that mediate ODV-binding seem to be host-range determinants, as resistance of *Spodoptera frugiperda* larvae to AcMNPV could partially be explained by differences in efficiencies in ODV-binding to the midgut, when compared to ODVs of Spodoptera frugiperda multiple nucleopolyhedrovirus (SfMNPV) [38]. This was further supported by an experiment, in which either PIF1 or 2 of Helicoverpa armigera nucleopolyhedrovirus (HearNPV) were replaced by their homologs from Spodoptera litura nucleopolyhedrovirus (SpltNPV). The resulting HearMNPV hybrids with the single *pif*-gene replacements were not orally infectious for *Helicoverpa armigera* larvae [39]. In contrast, a hybrid HearNPV, in which PIF3 was substituted for the SpltNPV homologue from, was able to infect *H. armigera* larvae. PIF3 is not involved in ODV binding and apparently, the biological function of PIF3 is (to a certain extend) inter exchangeable between these viruses [11]. On the other hand, PIF1 and 2 seemed to mediate ODV-binding species specifically. In these experiments, it has not been analysed whether the hybrid virus was able to form a complex. So, it remains to be established whether the failing binding properties of these PIFs affected oral infectivity or whether these PIFs are not compatible with the remaining native PIFs to form a functional entry complex.

### 5.4. PIF4 Provides Proteolytic Resistance to the Stable Core

PIF4 was identified when an AcMNPV *ac96* deletion mutant was not able to orally infect *Trichuplusia ni* larvae [12]. Similar results were obtained with HearMNPV and Bombyx mori NPV (BmNPV) after deletion of the *ac96* homologs *ha85* and *bm79* [40,41]. In AcMNPV, PIF4 was found in the ODV and BV envelopes by western blot analysis but this was not supported by mass spectrometry analysis of the virus particles [12,42,43,44]. However, PIF4 was detected by mass spectrometry after immune precipitation of PIF1, indicating that the complexity of the protein samples affected detection of this protein when analysing whole virus particles [4]. Nevertheless, the *pif4* deletion mutant of AcMNPV showed wild type levels of BV production and mortality in injection assays [12]. In ODVs of AcMNPV, PIF4 is essential for oral infectivity and is part of the core complex [4]. However, unlike *pif1*, *2* and *3*, deletion of the *pif4*-gene (*ac96*) did not affect interactions between the other components of the core complex as a smaller stable complex made-up of PIF1, 2 and 3 was found in the *pif4* deletion mutant.

This smaller complex was only found in ODVs that were released from OBs obtained from cell culture infections (C-OBs) and not in ODVs from larval-derived OBs (L-OBs) [20]. When analysing ODVs from L-OBs, the smaller complex appeared to be degraded by host-derived proteases that were co-occluded in the OBs. When these proteases were inactivated by heat treatment of L-OBs prior to their dissolution in alkaline buffer and isolation of the ODVs, the smaller complex was found again, like in ODVs from C-OBs. Apparently, the presence of PIF4 in the stable core provides the core complex resistance against proteolytic degradation by alkaline proteases.

Such a property of PIF4 would also explain the, at the time contradictory, results obtained earlier with HearNPV, where no smaller complex with PIF1, 2 and 3 was found after deletion of the *pif4*-homolog *ha85* [41]. As these analyses were performed with larval-derived ODVs, the smaller complex could have been degraded by host-derived proteases. Another contradictory finding was that HA85 was not detected in the stable core by western blot and that immunoprecipitation of HearMNPV PIF1 resulted in co-precipitation of P74, PIF2 and PIF3 but not HA85 [41]. To which extent the experimental conditions or the presence of host-derived proteases complicated these analyses is not clear. A yeast-two-hybrid assay nevertheless identified interactions between PIF4-P74, PIF4-PIF1, PIF4-PIF2 and PIF4-PIF3 [41]. Similar interactions of PIF4 with PIF1, PIF2 and PIF3 were also identified in BmNPV by bimolecular fluorescence protein complementation assays (BIFC) and co-immunoprecipitation studies after overexpression of these PIFs [40].

In conclusion, there is strong evidence that in addition to AcMNPV, other baculovirus species form a core complex with PIF1, PIF2, PIF3 and possibly also PIF4. However, the exact nature of the protein-protein interactions needs further validation under native conditions. Although PIF4 in AcMNPV appears not to be important for interactions between the remaining components of the stable core (PIF1-3), this PIF is required for the formation of the entire ODV entry complex as this complex was not detected in the ODV-envelope, released from C-OBs, of the *pif4* deletion mutant with blue-native PAGE [4]. These results suggest that PIF4 is important for establishing interactions between the stable core and the other associated PIFs and that these interactions are crucial for oral infectivity. The *pif4* deletion mutant has not been tested so-far in R18 de-quenching or live imaging assays.

## 6. PIFs That Associate with Lower Affinity to the Core Complex

### 6.1. PIF0 Mediates ODV Binding with the Midgut Epithelium

Historically, P74 was the first ODV-envelope protein that was found to be essential for oral infectivity and is now also named PIF0 [45]. This PIF-protein lacks an N-terminal signal sequence for routing to the inner nuclear membrane. Instead, PIF0 has two C-terminal transmembrane domains, which are essential for nuclear translocation of this protein (Figure 3) [46,47,48]. The Phobius-algorithm predicts that PIF0 is C-terminally anchored to the ODV envelope and that the N-terminus is directed outwards from the ODV-envelope, because of its non-cytoplasmic orientation in the host cell. Such a topology is in accordance with results from R18 de-quenching assays and live imaging of fluorescent ODVs that showed that PIF0 is important for ODV binding with midgut epithelial cells [35,49]. When ODVs lack PIF0, one third of these bind to the midgut epithelium, compared to wild type ODVs [49]. Once bound, about half of the bound ODVs fused with the epithelial membrane, just like wild type ODVs. This suggests that fusion is not affected by the absence of PIF0, as reasoned by Haas-Stapleton et al. 2004. Two independent studies showed that incorporation of PIF0 in the ODV-envelope seems not absolutely required to be able to play a role in oral infectivity [47,48]. The first study showed that co-feeding of purified PIF0-GFP fusion protein along with ODVs of *pif0* deletion mutant, rescued oral infectivity [48]. Similar results were obtained with purified C-terminal truncated variants of PIF0, lacking the transmembrane domains. The co-feeding of these soluble forms of PIF0 also rescued the oral infectivity of a *pif0* deletion mutant [47]. Apparently, the C-terminal transmembrane domains of PIF0 are dispensable for oral infectivity, while the rest of the protein mediates oral infectivity via interactions with other PIFs and likely also via interactions with host factors. Co-immunoprecipitation studies demonstrated that PIF0 interacts with PIF1 and PIF3 and further analysis revealed that PIF0 was a component of the entry complex, which was observed only under non-denaturing conditions [4,5]. Apparently, PIF0 associates with the core with relatively low affinity and might therefore be regarded as a more loosely associated component of the ODV entry complex than PIF1-4.

### 6.2. PIF0 Is Cleaved by Co-Occluded Proteases and Trypsins

**Figure 7:**
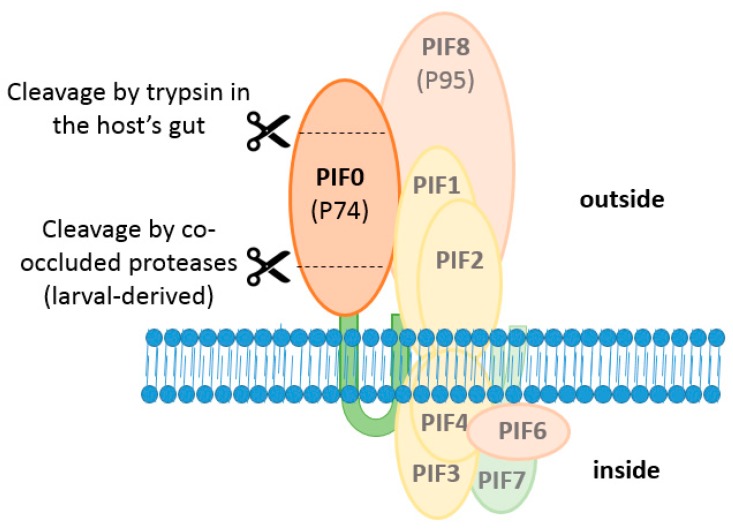
Two step cleavage model of PIF0. Firstly, PIF0 is cleaved by co-occluded host-derived proteases when larval-derived OBs dissolves in an alkaline environment. These 35 and 40 kDa fragments remain associated with the core complex. Secondly, a 20 kDa N-terminal fragment is cleaved off by trypsin in the host’s gut.

PIF0 is proteolytically cleaved in two separate events, once by a host-derived protease upon OB dissolution in the alkaline environment of the midgut and once by trypsins, present in the gut of the larva (Figure 7) [50]. In the first event, PIF0 is cleaved into two fragments of approximately 35 and 40 kDa. This cleavage was observed with ODVs released from L-OBs but not with ODVs from C-OBs, indicating that the protease was obtained from the larval host. After cleavage, both fragments remain associated with the core complex as these precipitated together with PIF1 in a co-immunoprecipitation study with PIF1 antiserum [50]. When the ODV entry complex lacks one of the components of the core complex, the cleaved form of PIF0 was not found but instead PIF0 was degraded. This suggests that the conformation of the ODV entry complex prevents degradation of PIF0, resulting in cleavage of this PIF [20]. However, the importance of this cleavage event for oral infectivity of ODVs is not clear as a bioassay with *T. ni* larvae showed that L-OBs and C-OBs resulted in a similar level of mortality, although the L-OBs killed these larvae slightly faster than C-OBs [51].

In the second cleavage event, a 20 kDa fragment is cleaved from the N-terminus of PIF0 by trypsins present in the midgut. This cleavage event was observed when purified GFP-tagged PIF0 was incubated with BBMV’s made from endothelial cells of third instar *Helicoverpa zea* or fourth instar *T. ni* larvae [52,53]. This trypsin-mediated cleavage appears important for oral infectivity as inhibition of cleavage by soybean trypsin inhibitor or through mutation of conserved arginine residues in the cleavage site affected the infectivity of ODVs [53]. However, the trypsin inhibitor might inhibit, in addition to PIF0, yet unidentified cleavage events of other ODV-envelope proteins that might also affect oral infectivity of ODVs. Furthermore, the effects of a mutated cleavage site on oral infectivity was determined by feeding isolated OBs to *T. ni* larvae, while the presence of the modified PIF0-molecules was only validated in transfected insect cells and not in the ODVs. So, when these mutations would affect proper routing of PIF0, as shown before with some PIF3 mutants, the decreased oral infectivity might be explained by absence of (mutated) PIF0 in the ODV envelope. However, trypsin-mediated cleavage of PIF0 was also observed in wild type ODVs, where this PIF associates with other PIFs in the entry complex. This indicates that this cleavage event also occurs under native conditions and might therefore be important for oral infectivity of ODVs [53].

### 6.3. PIF6 Is Also a Component of the ODV Entry Complex

The ODV-envelope protein AC68 was identified as PIF6, by Nie et al. [14], when a *lef3*-*ac68* double deletion bacmid was repaired with *lef3* and the resulting *ac68* deletion virus displayed the phenotype of the typical *pif* deletion mutant. PIF6 was not only found in the envelope of ODVs but also in BVs [14]. Its function in BVs is unknown, since BV production and infectivity were unaffected in absence of this protein. In ODVs, PIF6 precipitated together with PIF1 in a co-immunoprecipitation study with PIF1 antiserum and was identified as part of the entry complex under non-denaturing conditions [4,20]. So PIF6 appears to be important for ODV oral infectivity in the context of the entry complex. Predictions with the Phobius algorithm revealed this PIF does not have an INM-SM but instead has a C-terminal hydrophobic domain and a non-cytoplasmic oriented N-terminus (Figure 3). This indicates that PIF6 is probably directed outwards from the ODV-envelope. However, the biological importance of PIF6 for ODV binding, fusion or for a downstream process as hypothesized for PIF3, has yet to be determined. 

### 6.4. The Zinc-Finger Domain of PIF8 Is Important for Oral Infectivity

AC83 (also known as VP91 and P95) has two distinct roles during the infection cycle. Firstly, AC83 is required for nucleocapsid formation and is therefore essential for the production of BVs and ODVs [16]. Secondly, AC83 is involved in oral infectivity. The protein has a zinc-finger (ZF) domain (also addressed as chitin-binding domain (CBD)) and mutants that lack this domain show the phenotype of a *pif* deletion mutant. Although this protein has multiple roles in the infection cycle, it is considered to be a PIF and is now named PIF8 [9]. Oral infectivity of ODVs of such a mutant was partially rescued by feeding OBs to larvae together with Calcofluor white, an agent known to damage the peritrophic matrix [16]. A similar effect was not noted for viruses with a deletion of *p74*, *pif1*, *2* or *3* [54]. These data suggest that PIF8 binds to the chitin component of the peritrophic matrix. Affinity for chitin might also be advantageous for binding to the brush borders of epithelial cells as brush border microvilli produce chitin for the peritrophic matrix [55]. Possible interaction of PIF8 with host factors is also supported by the Phobius algorithm, which indicates that the topology of this protein is non-cytoplasmic, so is directed outwards from the ODV envelope after virion envelopment (Figure 1).

In the ODV envelope, PIF8 associates with the ODV entry complex with lower affinity than the components of the stable core, as this PIF was only found in the complex under non-denaturing conditions [4]. As this complex was not found in the mutant virus with the truncated form of PIF8, that lacks the ZF-domain, it was hypothesized that this domain is involved in formation of the entry complex by recruiting other PIFs [9]. However, this hypothesis was not experimentally supported as it was not determined whether the other PIFs (and the truncated PIF8 itself) were indeed absent in the envelope of mutant ODVs.

## 7. PIF5 Might Operate Independent from the ODV Entry Complex

ODV-E56 (AC148) has been established as PIF5 on the basis that deletion of the corresponding gene significantly impaired oral infectivity of ODVs [13,56]. However, low-level mortality has consistently been observed after deletion of this gene, suggesting that PIF5 is important but not crucial for oral infectivity, in contrast to the other PIFs. PIF5 has a C-terminal hydrophobic domain for translocation to the ODV envelope (Figure 3) [57]. When this domain was substituted for β-galactosidase, PIF5 translocated to the virogenic stroma and finally ended up in the nucleocapsids, instead of in the ODV envelope [57]. PIF5 was not detected in the entry complex and did not co-immune precipitate with PIF1 [4]. However, in a yeast-two-hybrid analysis PIF5 reciprocally interacted with PIF3, a component of the core complex [58]. Whether this interaction also occurs in the ODV envelope and in the presence of all other PIFs has yet to be determined. Like PIF3, PIF5 seems also not to be involved in ODV binding and fusion [56]. It could therefore be speculated that PIF5 mediates oral infectivity after binding and fusion, may be in association with PIF3. At this moment, the post-fusion configuration of PIF-proteins is entirely enigmatic though. It is presently also unknown whether the entry complex remains intact, falls apart, or even changes its composition after ODV binding and fusion.

## 8. ODV-E66 Might Retrospectively Be Assigned as PIF9

**Figure 8:**
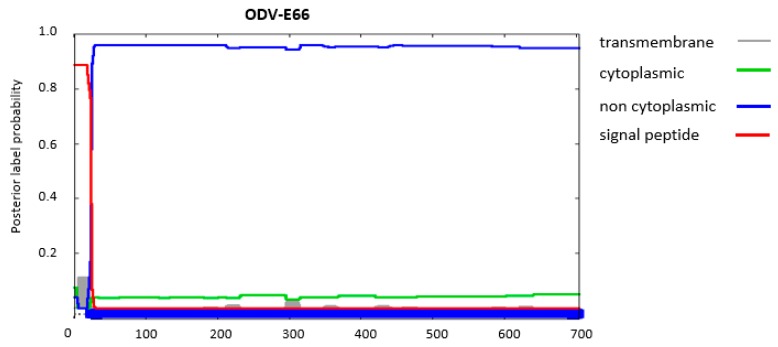
Prediction the presence of a transmembrane domain and signal peptides in ODV-E66 and orientation of this protein in an infected host-cell with the Phobius algorithm [17]. On the *y*-axis: the probability for the presence of transmembrane domains (grey), cytoplasmic (green) or non-cytoplasmic (**blue**) orientation of the sequence and a signal peptide (red). The final result of the prediction is summarized by the bar at the bottom with the indicated colours. The *x*-axis shows the number of the amino acids of that specific sequence.

ODV-E66 (*ac46*) is important but not crucial for midgut infection as deletion of this gene, like with PIF5, only impairs oral infectivity of ODVs [59,60]. This protein mediates oral infectivity possibly by (co-) facilitating ODV-binding with the midgut epithelium as peptide-derivatives of ODV-E66 compete with ODVs for binding with BBMVs [59]. These results are in accordance with predictions of Phobius-software that indicates that ODV-E66 is N-terminally anchored in the ODV envelope by its INM-SM and that the rest of the protein directed outwards from the ODV envelope, because of its non-cytoplasmic topology in the host-cell, enabling interactions with host factors (Figure 8). Two studies indicated that ODV-E66 interacts with other components of the ODV entry complex. In a yeast-two-hybrid analysis, ODV-E66 reciprocally interacted with PIF2 and PIF3 [58]. Furthermore, in co-immunoprecipitation studies and bimolecular fluorescence protein complementation assays with BmNPV, ODV-E66 interacted with the PIF4 homolog BM79 [40]. In light of this, it is interesting to determine whether ODV-E66 associates with the ODV entry complex. As ODV-E66 appears to be important (but not crucial) for oral infectivity by (co-) facilitating ODV-binding with the midgut epithelium, the protein can retrospectively be assigned as PIF9.

## 9. Conclusions and Perspectives

The infection cycle of baculoviruses starts with infection of midgut epithelial cells. To mediate this infection, the ODV envelope contains an array of at least ten different PIFs (including PIF9). The genes that encode these proteins are highly conserved among all baculoviruses and, with exception of *ac110* (encoding PIF7), in other large, nuclear replicating invertebrate DNA viruses (Reviewed by Wang et al. [10]). The fact that the *pif*-genes are found in a large group of evolutionary related invertebrate DNA viruses that have co-evolved with their hosts for millions of years, suggest that PIF-mediated entry is a commonly used and ancient entry mechanism [61]. The number of PIFs seems to increase gradually during evolution, from PIF0-PIF3 in the *Nimaviridae* to all ten in the *Baculoviridae*, suggesting that PIF-mediated entry became more and more advanced over time [10]. As said, ten different proteins are known to be involved in midgut infection by baculovirus ODVs but there are maybe even more. A good candidate is AC108 that precipitated together with PIF1 [4].

Furthermore, eight out of ten PIFs form a large complex in the ODV envelope, suggesting that PIFs cooperatively mediate cell entry in a network of protein-protein interactions. This finding complicates the interpretation of studies with *pif*-deletion mutants as the observed phenotypes concerning ODV binding and fusion cannot be directly attributed to absence of a PIF-protein itself but may be a consequence of indirect effects because of lost protein-protein interactions. Hence, making deletion mutants is a good approach to identify PIFs but not to elucidate the molecular mechanism of PIF-mediated cell entry or to identify the biological importance of a single PIF in that process. Now that we know that there are many PIF-proteins of which at least eight form a complex, the questions that emerge are: what is the role of the ODV entry complex during midgut infection and do the various PIFs have different functions?

In absence of PIF3, a crucial protein for complex formation, ODVs are still able to bind and fuse with the plasma membrane of columnar cell brush borders but nevertheless fail to infect the larval midgut. This suggests that the entry complex itself is not crucial for binding and fusion but probably mediates infection by serving as a scaffold for proteins that mediate binding and fusion (PIF0, PIF1, PIF2 and maybe also PIF9) and proteins involved in downstream processes (such as PIF3). However, what that these downstream processes are and what other function(s) PIFs have besides mediating binding and fusion during midgut infection is not clear.

Normally, the midgut epithelium digests and absorbs nutrients. Viruses may exploit this physiology for entry and passage of the midgut barrier. For example, the *Jujonia coenia* densovirus is transported across the midgut epithelium of *S. frugiperda* larvae via transcytosis, which is normally used to transport large unprocessed proteins [62,63]. Furthermore, infection with this densovirus altered the structural and functional properties of the epithelial tissue is such a way that it negatively affected its gate function. However, how infection with baculoviruses affects the midgut physiology and how PIFs are involved in this is unexplored. These insights might be important to define what other processes besides ODV binding and fusion are required for successful infection of the midgut epithelium.

## Figures and Tables

**Table 1:** Identified PIFs in AcMNPV.

PIF	ORF	Size (kDa)	Nr. Of Cys.	Reference
PIF0 (P74)	*ac138*	74	6	Faulkner et al. [6]
PIF1	*ac119*	60	24	Kikhno et al. [7]
PIF2	*ac22*	44	14	Pijlman et al. [8]
PIF3	*ac115*	23	12	Ohkawa et al. [11]
PIF4	*ac96*	20	2	Fang et al. [12]
PIF5 (ODV-E56)	*ac148*	56	6	Harrison et al. [13]
PIF6	*ac68*	16	1	Nie et al. [14]
PIF7	*ac110*	7	1	Liu et al. [15]
PIF8 (VP91/P95)	*ac83*	96	13	Zhu et al. [16]
